# Glycan Biosynthesis Ability of Gut Microbiota Increased in Primary Hypertension Patients Taking Antihypertension Medications and Potentially Promoted by Macrophage-Adenosine Monophosphate-Activated Protein Kinase

**DOI:** 10.3389/fmicb.2021.719599

**Published:** 2021-11-04

**Authors:** Shuai Zheng, Chunmei Piao, Yan Liu, Xuxia Liu, Tingting Liu, Xiaoping Zhang, Jingyuan Ren, Yulei Liu, Baoli Zhu, Jie Du

**Affiliations:** ^1^Beijing Anzhen Hospital, Capital Medical University, Beijing, China; ^2^Beijing Collaborative Innovation Centre for Cardiovascular Disorders, Beijing, China; ^3^The Key Laboratory of Remodeling-Related Cardiovascular Diseases, Ministry of Education, Beijing Institute of Heart Lung and Blood Vessel Diseases, Beijing, China; ^4^Department of Hypertension, Beijing Anzhen Hospital, Capital Medical University, Beijing, China; ^5^Department of Clinic Laboratory, Beijing Anzhen Hospital, Capital Medical University, Beijing, China; ^6^CAS Key Laboratory of Pathogenic Microbiology and Immunology, Institute of Microbiology, Chinese Academy of Sciences, Beijing, China; ^7^Collaborative Innovation Center for Diagnosis and Treatment of Infectious Diseases, The First Affiliated Hospital, College of Medicine, Zhejiang University, Hangzhou, China

**Keywords:** gut microbiota, primary hypertension, medication, macrophage, AMP-activated protein kinase, glycan

## Abstract

Increasing evidences suggest that the gut microbiota have their contributions to the hypertension, but the metagenomic characteristics and potential regulating mechanisms in primary hypertension patients taking antihypertension drugs are not clear yet. We carried out a metagenomic analysis in 30 primary hypertension patients taking antihypertension medications and eight healthy adults without any medication. We found that bacterial strains from species, such as *Bacteroides fragilis*, *Bacteroides vulgatus*, *Escherichia coli*, *Klebsiella pneumoniae*, and *Streptococcus vestibularis*, were highly increased in patients; and these strains were reported to generate glycan, short-chain fatty acid (SCFA) and trimethylamine (TMA) or be opportunistic pathogens. Meanwhile, *Dorea longicatena*, *Eubacterium hallii*, *Clostridium leptum*, *Faecalibacterium prausnitzii*, and some other strains were greatly decreased in the patient group. The Kyoto Encyclopedia of Genes and Genomes (KEGG) analysis found that ortholog groups and pathways related to glycan biosynthesis and multidrug resistance were significantly increased in the patient group, and some of the hub genes related to *N*-glycan biosynthesis were increased in the patient group, while those related to TMA precursor metabolism and amino acid metabolism both increased and decreased in the patient group. Metabolites tested by untargeted liquid chromatography–mass spectrometry (LC-MS) proved the decrease of acetic acid, choline, betaine, and several amino acids in patients’ fecal samples. Moreover, meta-analysis of recent studies found that almost all patients were taking at least one kind of drugs that were reported to regulate adenosine monophosphate-activated protein kinase (AMPK) pathway, so we further investigated if AMPK regulated the metagenomic changes by using angiotensin II-induced mouse hypertensive model on wild-type and macrophage-specific AMPK-knockout mice. We found that the changes in *E. coli* and *Dorea* and glycan biosynthesis-related orthologs and pathways were similar in our cohort and hypertensive wild-type mice but reversed after AMPK knockout. These results suggest that the gut microbiota-derived glycan, SCFA, TMA, and some other metabolites change in medication-taking primary hypertension patients and that medications might promote gut microbiota glycan biosynthesis through activating macrophage-AMPK.

## Introduction

Hypertension is an increasing epidemic disease worldwide. In China, about 23% of the adult population had hypertension (Wang et al., 2018). Among multiple factors that affect blood pressure (BP) and hypertension, gut microbiota is regarded as a new regulator in recent years. Studies on animal models demonstrated that gut microbiota dysbiosis caused by antibiotics could upregulate BP (Pluznick et al., 2013), and angiotensin II (AngII) needed gut microbiota existence to induce hypertension (Karbach et al., 2016). It is known that some gut microbiota-derived metabolites could regulate BP. For instance, the short-chain fatty acids (SCFAs), especially acetate, propionate, and butyrate, could mainly reduce BP and inhibit hypertension (Pluznick et al., 2013; Marques et al., 2017; Wang et al., 2017). Meanwhile, trimethylamine (TMA) is reported to inhibit vascular smooth muscle cell viability (Jaworska et al., 2020), while trimethylamine *N*-oxide (TMAO) is reported to induce vascular endothelial cell injury (Chen et al., 2017), so as to promote hypertension. In human cohort studies, gut microbiota showed different enterotypes in healthy adults and hypertension patients (Li et al., 2017), and participants with lower BP had higher abundances of several SCFA-producing microbes (Verhaar et al., 2020), while circulating TMAO showed a dose–response relationship with increased odds of hypertension (Abbasalizad Farhangi and Vajdi, 2020). Meanwhile, gut microbiota and its metabolites also participate in other hypertension-related cardiovascular diseases, such as coronary artery disease (Kazemian et al., 2020). Considering the complicated functions of gut microbiota, it is important to make comprehensive cognition of gut microbiota characteristics under hypertension, so as to better understand the pathological mechanisms of hypertension development and adopt better clinical interventions for treating hypertension and related cardiovascular diseases.

Since gut microbiota could be altered by environmental factors, such as diet components and drugs/xenobiotics (Ursell et al., 2014), it is reasonable to hypothesize that hypertension patients who are taking antihypertension medications may have unique gut microbiota structure and functions. In the past few years, the major part of studies on hypertension patients gut microbiota was carried out in pre-hypertension people and newly diagnosed hypertension patients prior to antihypertension treatment, and a few studies referring gut microbiota differences between healthy adults and antihypertension treated and non-treated patients only used 16S rDNA sequencing (Li et al., 2019); thus, the comprehensive changes of gut microbiota under antihypertension medications influences are not clear. In this study, we carried out metagenomic sequencing on fecal DNA samples of primary hypertension patients who were receiving antihypertension medications, and we analyzed both gut taxonomy and functional genes/pathways changes by comparing them with those of healthy control. Moreover, we found that almost all the investigated patients were taking at least one kind of drugs that were involved in regulating adenosine monophosphate-activated protein kinase (AMPK) pathway (and almost all of the drugs were reported to activate AMPK pathway; see Supplementary Tables 1, 2 for detailed information). Since AMPK pathway is essential for regulating macrophage phenotype and functions (Sag et al., 2008), and macrophage could regulate gut microbiota colonization (Earley et al., 2018), there is probability that the antihypertension medications could commonly (at least partially) regulate gut microbiota through macrophage-AMPK mechanism. To test such probability, we further used wild-type and macrophage-specific AMPK-knockout mice to make AngII-induced hypertension model, and we compared the intersection of metagenomic changes in mice and hypertension (HBP) patients, so as to uncover the potential macrophage-AMPK-regulated metagenomic components in the investigated HBP patients.

## Materials and Methods

### Human Cohort

The human cohort study protocol was reviewed and approved by the Clinical Research Ethics Committee of Anzhen Hospital. For this study, a cohort of 10 healthy volunteers and 31 patients were recruited. The 10 volunteers are staff and graduating students in Anzhen Hospital, and they did not take any medications at least 4 weeks before fecal sample collection. All 31 patients were inpatients of the Department of Hypertension during October 10, 2019, to November 15, 2019, in Anzhen Hospital. All patients were diagnosed as primary hypertension according to the previous medical histories and received antihypertension medications at least 1 day before fecal sample collection. Treatment effect of controlling BP was decided according to the guideline [generally reducing office BP to 140/90 mmHg, for 65− to 79-year-old patients, systolic BP (SBP) < 150 mmHg] (Joint Committee for Guideline Revision, 2019). And patients were allowed to be discharged from the hospital after they were in stable conditions (except for one patient, HBP29, who was discharged at her insistence). Individuals were excluded if they received antibiotics or probiotics within 4 weeks before being enrolled in the study. The basic physiological characteristics and clinical parameters of the health volunteers were based on their most recent physical examination reports in 2019, and these records of the patients were collected from the Clinical Data Center of Anzhen Hospital. According to 2020 International Society of Hypertension (ISH) guideline (Unger et al., 2020), healthy control is defined as SBP ≤ 130 mmHg and diastolic BP (DBP) ≤ 85 mmHg for untreated individuals, and hypertension is defined as current SBP ≥ 140 mmHg and/or DBP ≥ 90 mmHg for patients with or without antihypertension treatments. One volunteer was excluded after fecal sample collection, for his SBP was found higher than 140 mmHg for two continuous days after sample collection, and one volunteer and one patient were excluded for incomplete clinical information. There were finally eight healthy control and 30 patients for metagenomic comparison.

### Cohort Fecal Sample Collection and DNA Extraction

Fecal samples of healthy volunteers were collected freshly by themselves in the lab and immediately stored in −80°C freezer. And fecal samples of patients were collected from the Department of Clinic Laboratory of Anzhen Hospital after routine stool exam; all the samples were kept in room temperature for less than 4 h and immediately stored in −80°C freezer after collection. The routine stool exams were carried out approximately 2∼3 days after the patients were admitted and treated in the Department of Hypertension. All fecal sample DNA was extracted by using QIAamp DNA kit (QIAGEN Corp., Valencia, CA, United States) according to the manufacturer’s protocol.

### Generation of Knockout Mice and Hypertension Model

The animal study protocols were approved by the Ethics Committee of Anzhen Hospital. The generation of macrophage-specific AMPK-knockout mice was described in our previous publication (Zheng et al., 2019). Generally, we mated C57 background mice, which had *loxP* sites in AMPKα1 catalytic domain (AMPKα1^fl/fl^), with C57 background mice, which had colony-stimulating factor 1 receptor (Csf1r) promoter-driven expressed estrogen receptor-Cre fusion protein gene (Csf1r-Mer-iCre-Mer), and we obtained AMPKα1^fl/fl^/Csf1r-MerCre and AMPKα1^fl/fl^/WT mice. To induce AMPK knockout in macrophage, we intraperitoneally injected tamoxifen (Sigma-Aldrich Corp., St. Louis, MO, United States; 20 mg/ml in oil) to both phenotype mice for five continuous days (one time per day, 2 mg/day), and we identified the knockout effect by testing AMPKα1 mRNA in bone marrow cells (results were also in the publication, Zheng et al., 2019).

To generate hypertension model, we subcutaneously implanted osmotic minipumps to AMPKα1^fl/fl^/Csf1r-MerCre and AMPKα1^fl/fl^/WT mice, and we treated mice with AngII for 7 days at a pressor dose of 1,500 ng⋅kg^–1^⋅min^–1^. Five male mice of each genotype were used for the study. All the mice were pretreated with tamoxifen for 5 days starting at 8 weeks of age; they were mixed fed in two cages for 5 days to let Cre enzyme work and to obtain similar gut microbiota basal level; and then they were separately fed by genotype, and minipump was implanted. The mice also obtained further tamoxifen injection for the first 3 days after minipump implantation. BP was measured by tail-cuff plethysmography before minipump implantation and on day 7 of AngII stimulation.

### Mouse Fecal Sample Collection and DNA Extraction

Mouse fecal samples were collected in sterile hood by giving mice abdominal massage, and fresh fecal samples were collected into sterile Eppendorf tubes and kept in ice bath and stored at −80°C. We collected mouse fecal samples at two time points along with measuring BP: (1) after 5 days’ tamoxifen treatment while before minipump implantation and (2) on the day 7 of AngII stimulation. For each mouse, at least 0.2 g of fecal sample at each time point was collected. Mouse fecal DNA was extracted by StoolGen DNA kit (CW2092, Beijing CoWin Bioscience Co., Ltd, Beijing, China) following the manufacturer’s protocol.

### Metagenomic Sequencing and Data Processing

Fecal DNA concentration was evaluated by NanoDrop spectrophotometer, DNA quality was tested by agarose gel electrophoresis, and DNA sequencing was carried out on Illumina HiSeq X Ten System (Illumina, Inc., San Diego, CA, United States). DNA library was firstly constructed according to the manufacturer’s instructions, and paired-end libraries with an insert length of approximately 350 bp were built and sequenced from both ends with a read length of 150 bp. The raw reads were filtered by removing adaptor sequences (overlap > 15 bp), low-quality reads (N bases > 10 bp or low-quality bases > 40 bp), and host genome sequence contamination [aligned by SoapAligner (Li et al., 2009), parameters: identity ≥ 90%, –l 30, –v 7, -M 4, –m 200, –x 400]; and finally clean data were obtained.

For bioinformatics analysis, clean data of each sample were further assembled by SOAPdenovo (Luo et al., 2012) (parameters: k-mer 55, -d1, -M3, -R, -u, -F) to obtain Scaffolds and then Scaftigs (i.e., continuous sequences within Scaffolds), and unused reads from each sample’s clean data were mixed and assembled by the same parameter to obtain mixed Scaftigs. Then we used Scaftigs ≥ 500 bp to predict open reading frame (ORF) genes by MetaGeneMark (Li et al., 2014), and we filtered out gene < 100 nt. After filtering, the reversed ORF genes were clustered by CD-HIT (Fu et al., 2012) to construct non-redundant gene catalog (parameters: -c 0.95, -G 0, -aS 0.9, -g 1, -d 0), using 95% sequencing identity cutoff and 90% minimum coverage cutoff for the construction. Then the clean data of each sample were realigned to such gene catalog by SoapAligner (parameters: -m 200, -x 400, identity ≥ 95%), genes with ≥2 reads number were retained as Unigenes, and their reads numbers were used for calculating the abundances of genes.

Illumina sequence data of human cohort and mouse model reported in the paper was deposited in the National Center for Biotechnology Information (NCBI) Sequence Read Archive (SRA) database under bioproject number PRJNA685581^1^ (biosample numbers are from SAMN17132978 to SAMN17133037).

### Bioinformatics Analysis

To obtain taxonomic information, the Unigenes were aligned to the NCBI NR database (Version: 2014-10-19) by DIAMOND (Buchfink et al., 2015) (default parameters, evalue ≤ 0.00001) and annotated to bacteria, fungi, archaea, and virus taxonomic groups; then we retained genes by evalue ≤ 10 ^∗^ top hit evalue as significant matching genes; and we used these genes to distinguish taxonomic groups by MEGAN (Huson et al., 2007). The abundance of each taxonomic group was determined by summing the abundances of genes in this group.

To obtain functional information, the Unigenes were aligned to Kyoto Encyclopedia of Genes and Genomes (KEGG) database (Kanehisa et al., 2008) by DIAMOND (also used default parameters, evalue ≤ 0.00001) and assigned to orthologs, different levels of pathways, and modules by retaining the one High-scoring Segment Pair (HSP) > 60 bits of annotated hits. The abundance of ortholog group/pathway/module was determined by summing the abundances of genes assigned in this feature.

To assess bacterial richness, α-diversity was evaluated using Shannon index, Simpson index, and Evenness index; and β-diversity was evaluated by principal component analysis (PCA). Both α- and β-diversity were based on abundances of genus, species, and KEGG ortholog groups.

### Untargeted Liquid Chromatography–Mass Spectrometry Analysis

Metabolites in fecal and plasma samples of study cohort were evaluated by untargeted liquid chromatography–mass spectrometry (LC-MS) analysis carried out in Fan-Xing Biological Technology Co., Ltd, Beijing, China.

For sample preparation, a mixture of acetonitrile/methanol (75:25 v/v, 300 μl) was added to the fecal (100 μl/100 mg) and plasma for protein deposition. After vortexing for 60 s, the mixture was left to rest for 10 min and centrifuged at 12,000 rpm for 10 min at 4°C. Then the supernatant was filtered by syringe filters (0.22 mm, Jinteng, Tianjin, China) prior to LC/MS/MS analysis. The Nexera X2 system (Shimadzu, Kyoto, Japan) and Triple TOF 5600 quadrupole–time-of-flight mass spectrometers (AB Sciex, Redwood City, CA, United States) were used to analyze the ultra-performance LC combined with quadrupole time-of-flight tandem MS (UPLC Q-TOF MS/MS).

LC separation was performed on ZORBAX Eclipse Plus C18, column (2.1 mm × 100 mm × 3.5 μm, Agilent Technologies, Santa Clara, CA, United States). The column was maintained at 45°C. The injected sample volume was 10 μl for each run in the full loop injection mode. The flow rate of the mobile phase was 0.5 ml/min. In reversed-phase LC (RPLC) mode, gradient elution was performed with the following solvent system: (A) 0.1% formic acid–water and (B) acetonitrile with 0.1% formic acid. The gradient started with 98% A and decreased to 10% A in 13 min, holding at 10% A for 3 min, then turned to 98% A immediately, and holding at 98% A for 4 min.

Mass spectrometry experiments were performed on Triple TOF 5600 + an orthogonal accelerated TOF MS (AB Sciex, United States) equipped with an electrospray ion source. Data were acquired in both positive and negative V-geometry modes for each chromatography separation technique LC-MS analysis. The capillary voltages were set to 2,500 and 3,000 V, respectively. Other settings are as follows: cone gas 50 L/h, desolvation gas 600 L/h, source temperature 120°C, and desolvation temperature 500°C. The scan range was from *m*/*z* 50 to 1500 in the full scan mode, and data were collected in centroid mode. Independent reference lock-mass ions via the Analyst TF 1.6 and MarkerView 1.2.1 were used to ensure mass accuracy during data acquisition.

The raw data from LC-MS were analyzed by MS-DIAL software for peak detection, peak filtration, and peak alignment and generated original data table including *m*/*z* and peak area information. Then MetaboAnalyst 4.0^2^ was used to normalize the original data and carry out further analysis. Compounds between two groups were compared by two-tailed Welch’s *t*-test based on their peak intensities, and *p*-value < 0.05 was considered statistically significant. Partial least squares discrimination analysis (PLS-DA) was also carried out by MetaboAnalyst, and variable importance in projection (VIP) value for each compound was decided. Fold change (FC) of each compound from control group to the patient group was also evaluated. Compounds with VIP > 1 and *p* < 0.05 were decided as significantly different between the two groups. All compounds with VIP > 1 were assigned to metabolites by searching in the Human Metabolome Database (HMDB)^3^ (Wishart et al., 2009); the mass tolerance for the HMDB search was set at 0.05 Da. And identified metabolites were then assigned to metabolomics pathways according to HMDB, KEGG, and Metlin^4^ by MetaboAnalyst.

### Statistical Analysis

Comparison of cohort physiological index, α-diversity index, bacterial abundance, and KEGG ortholog/pathway/module abundances were by the Mann–Whitney two-tailed test, unless otherwise stated. Association of age/fasting blood glucose (FBG)/triglyceride (TG)/high-density lipoprotein (HDL) with some greatly changed bacterial strains and gut microbiome orthologs and pathways were calculated by Spearman’s correlation test (two-tailed). *p* < 0.05 was taken to indicate statistical significance for each test. All analyses were carried out using GraphPad Prism version 5.0 for Windows (GraphPad Software Inc., La Jolla, CA, United States).

## Results

### Hypertension Patients Had Similar Gut Microbiome to Healthy Control

We analyzed gut metagenomic structures of eight healthy volunteers (control group, Ctrl) and 30 patients (HBP group, HBP). The detailed physiological parameters and medication records of the cohort are shown in Supplementary Table 1, and statistical comparisons of basic physiological index between Ctrl and HBP groups are shown in Table 1. We can see that other than SBP and diastolic BP (DBP), the FBG, TG, and HDL levels were also significantly different between healthy control and HBP patients. All the drugs involved in the medication records were searched in literature for their effects on AMPK pathway, and the related reports are summarized in Supplementary Table 2. All the 38 fecal DNA samples were sequenced on the Illumina HiSeq platform and generated 484.3 Gb of 150-bp paired-end reads, with an average of 12.7 Gb per sample. After filtering, the clean data were assembled into Scaffolds and further generated Scaftigs for gene prediction, taxonomic classification, and functional annotation. The sequencing data and final Scaftigs information are summarized in Supplementary Table 3.

**TABLE 1:** The basic physiological index of study cohort.

Characteristics	Ctrl	HBP	*p*-Value
Number	8	30	/
Gender (% female)	50	36.7	/
Age, year	34.5 ± 7.2	51 ± 14.3	<0.05
SBP, mmHg	118.4 ± 7.2	138.8 ± 12.8	<0.01
DBP, mmHg	73.3 ± 8.0	88.0 ± 11.9	<0.01
FBG, mmol/L	4.96 ± 0.37	5.66 ± 1.16	<0.05
TC, mmol/L	4.18 ± 0.48	4.84 ± 1.17	0.09
TG, mmol/L	0.89 ± 0.21	2.10 ± 1.27	<0.01
HDL, mmol/L	1.41 ± 0.22	1.12 ± 0.24	<0.01
LDL, mmol/L	2.54 ± 0.48	2.86 ± 0.70	0.25

*The average level of related physiological index in healthy control and hypertension patient groups is shown here. Data are mean ± SD (standard deviation). *p*-Values are evaluated by Mann–Whitney two-tailed test. Blood pressure of HBP group was the recorded on fecal sample collection days.*

*Ctrl, control; HBP, hypertension; SBP, systolic blood pressure; DBP, diastolic blood pressure; FBG, fasting blood glucose; TC, total cholesterol; TG, triglyceride; HDL, high-density lipoprotein; LDL, low-density lipoprotein.*

**FIGURE 1:**
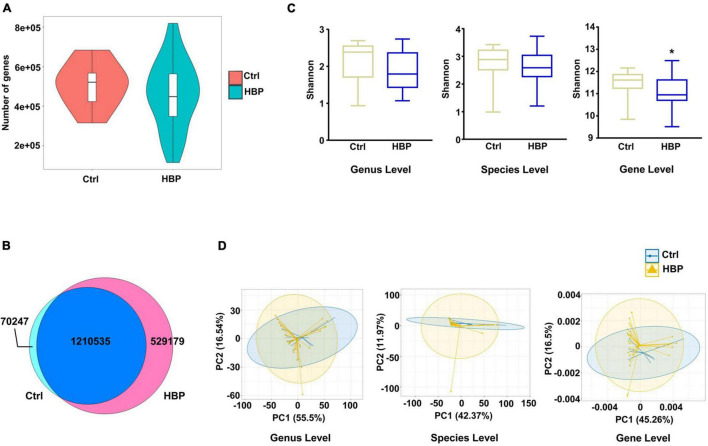
Microbial diversity shows slight difference between healthy control and primary hypertension patients with antihypertension medications. **(A)** Gene count comparison between the two groups. **(B)** Venn comparison of the shared gene numbers and unique gene numbers between the two groups. **(C)** α-Diversity analysis (Shannon index) based on genus, species, and ortholog profile in the two groups. **(D)** Principal component analysis (PCA) of β-diversity analysis based on genus, species, and orthologs profile between the two groups. Ctrl, healthy control group, *n* = 8. HBP, primary hypertension patient group who took antihypertension medications before testing metagenomics, *n* = 30. Gene level, i.e., ortholog level. *p < 0.05 by one-tailed Mann–Whitney *U*-test.

We first compared the gene numbers and category differences between healthy control and HBP patients, and we found that the HBP group had a little lower average gene number than Ctrl (Figure 1A), and the two groups shared over 70% of the gene categories (Figure 1B). Bacterial richness was reported to be greatly decreased in HBP patients in previous report (Li et al., 2017), so we also evaluated the diversity difference based on genus, species, and ortholog profile. As shown in Figure 1C, the α-diversity calculated by Shannon index was lower in the HBP group, but only reached statistical significance at ortholog level. Meanwhile, we also calculated α-diversity by Simpson and Evenness indexes, and these two indexes were also lower in the HBP group at all three levels but did not reach statistical significance (Supplementary Figure 1). And β-diversity evaluated by PCA based on genus, species, and orthologs profile showed little difference between the two groups (Figure 1D). These results suggest that the HBP patients have similar gut microbiome as in healthy control.

### Abundances of Gut Microbiota Strains Related to Glycan, Short-Chain Fatty Acid, and Trimethylamine Metabolism Greatly Changed in Hypertension Patients

**FIGURE 2:**
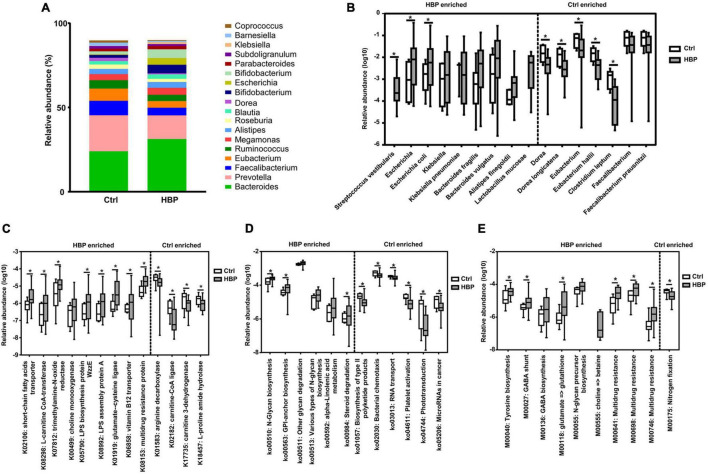
Microbiome abundances at different levels show significant differences between healthy control and primary hypertension patients with antihypertension medications. **(A)** The relative abundances of the top 18 highest abundant genera in the two groups. **(B)** The most significantly different genera and species and their relative abundances between the two groups. Nine strains enriched in patient group are shown on the left side, and seven strains enriched in control group are shown on the right side. **(C)** Part of the most significantly different Kyoto Encyclopedia of Genes and Genomes (KEGG) ortholog groups and their relative abundances between the two groups. **(D)** The most significantly different KEGG pathways and their relative abundances between the two groups. **(E)** The most significantly different KEGG modules and their relative abundances between the two groups. Categories enriched in control group and patient group are separately shown on the right side and left side of each graph. *p < 0.05 by two-tailed Mann–Whitney *U*-test. Ctrl, healthy control group. HBP, primary hypertension patient group who took antihypertension medications.

Then we investigated the abundant changes of gut microbiota from Ctrl to HBP group. The relative abundances of all identified bacterial taxonomies in each individual are summarized in Supplementary Table 4. Figure 2A shows the abundant variations of the top 18 highest abundant genera from Ctrl to HBP group, and the changes were obvious in genera such as *Bacteroides* and *Faecalibacterium*. Statistical analysis found a series of bacterial strains that had significant abundant differences between Ctrl and HBP groups, and Figure 2B shows the genus and species among these strains (including some strains with a trend to be significant). Specifically, the SCFA-generating bacteria *Bacteroides fragilis*, *Bacteroides vulgatus*, and *Alistipes finegoldii* under *Bacteroidetes* phylum (Yu et al., 2013; Guilin et al., 2020; Su et al., 2020) were enriched in the HBP group, while *B. fragilis* and some other members of the *Bacteroidetes* are also major bacterial producers of glycan (Coyne et al., 2008; Martens et al., 2009). Meanwhile, the stains from Enterobacteriales–Enterobacteriaceae branch under Proteobacteria phylum were also overexpressed in the HBP group, including *Escherichia* (*Escherichia coli*), *Klebsiella* (*Klebsiella pneumoniae*), and *Citrobacter*; and these bacteria are reported to generate TMA (Kalnins et al., 2015; Rath et al., 2017; Zeisel and Warrier, 2017). Besides, *Lactobacillus mucosae* and *Streptococcus vestibularis* under Firmicutes phylum were increased in the HBP group as well: the former species has genes mainly reflected in carbohydrate metabolism (Jia et al., 2020), and the latter one is reported as oral-enriched bacteria and could opportunistically cause infection (Tufan et al., 2010).

By contrast, some strains from Clostridia–Clostridiales branch under Firmicutes phylum were enriched in Ctrl. These bacteria include *Dorea* (*Dorea longicatena*), *Eubacterium* (*Eubacterium hallii*), *Clostridium leptum*, and *Faecalibacterium* (*Faecalibacterium prausnitzii*) (Figure 2B, right side). *Dorea* is reported to produce butyrate (Yang et al., 2019); *Eubacterium* is reported to convert methanol into *n*-butyrate (Huang et al., 2020) and to generate TMA (Rath et al., 2017); and *C. leptum* and *F. prausnitzii* are also reported to be butyrate producers (Kang et al., 2018; Nilsen et al., 2020). Taken together, the HBP group had some elevated bacteria concerning glycan, SCFA, and TMA production, while healthy Ctrl had some other elevated bacteria, which mainly produce butyrate.

### Abundances of Metagenomic Functional Categories Related to Glycan, Short-Chain Fatty Acid, Trimethylamine Precursor, Amino Acid Metabolism, and Drug Resistance Greatly Changed in Hypertension Patients

Bacterial abundant differences suggested some functional changes in hypertension gut microbiota; thus, we aligned Unigenes to KEGG database (Kanehisa et al., 2008); we annotated them to ortholog groups, three levels of pathways, and Module groups; and we compared the differences of their relative abundances between HBP patients and healthy control. The related abundances of orthologs, third-level pathways, and Modules in each individual are respectively summarized in Supplementary Tables 5, 7, 8. Then we carried out the Mann–Whitney two-tailed test to compare the relative abundance of each microbiome component between the healthy Ctrl and HBP groups, then sorted them by *p*-value from the smallest to biggest, and decided whether the significantly different component was related to hypertension or other cardiovascular diseases based on its function and related reports.

Statistical analysis found that there were 38 ortholog groups significantly (or almost significant) enriched in the HBP group (summarized in Supplementary Table 6), and there were four ortholog groups significantly enriched in healthy control. Specifically, the HBP-group enriched ortholog groups are related to right functional aspects, including the following: (1) SCFA biosynthesis (K02106); (2) bioconversion of TMA precursors (K00499, K07812, and K08298); (3) glucose metabolism and utility (K00036, K00118, K08194, and K03670); (4) fatty acid metabolism (K13770); (5) lipopolysaccharide (LPS) biosynthesis (K05790 and K08992); (6) flagellum biosynthesis (K02399); (7) amino acids (including glycine, glutamate, glutathione, and serine) and vitamin B12 metabolism (K00281, K01580, etc.); and (8) multidrug resistance (K03543, K03923, etc.). Nine representative ortholog groups of the 38 groups are shown in Figure 2C, left side. The four healthy-group enriched orthologs include two enzyme genes for carnitine (also one precursor of TMA) metabolism (K02182 and K17735) and two enzyme genes for arginine and proline metabolism (K01583 and K18457), as shown in Figure 2C, right side. These results suggested that the gut microbiota in the HBP group adapted to the medication stimulation and had increased capabilities on generating SCFA and LPS and metabolizing TMA precursors/glucose/fatty acids/amino acids, while the gut microbiota in Ctrl were more capable of metabolizing arginine and proline. These ortholog changes were in accordance with gut microbiota taxonomic changes. As for carnitine bioconversion, three related metabolic genes were respectively enriched in the healthy control and HBP groups.

For the third-level KEGG pathway analysis, we found six pathways significantly enriched in the HBP group (three of them were almost significant), and four of them were around glycan biosynthesis and metabolism; the other two pathways were around lipid (ko00592) and steroid (ko00984) metabolism (Figure 2D, left side). Meanwhile Ctrl also had six significantly enriched KEGG pathways, as shown in Figure 2D, right side, but they were related to six different biological functions, such as polyketide production (ko01057) and bacterial chemotaxis (ko02030). These results suggested that the glycan bioconversion was the most enhanced function in HBP gut microbiota under medications.

For the KEGG module analysis, we found nine modules significantly enriched in the HBP group (three of them were almost significant). Among the nine modules, two of them were for *N*-glycan (M00055) and betaine (M00555) biosynthesis, respectively; and four of them were involved in amino acid metabolism (M00040, M00027, M00136, and M00118) for tyrosine, gamma-aminobutyrate, and glutamate; and the remaining three modules were for multidrug resistance (M00641, M00698, and M00746). On the other side, only one module for nitrogen fixation (M00175) was significantly enriched in healthy control. Such module changes are shown in Figure 2E. The module changes once again suggested that HBP gut microbiota had enhanced functions in glycan, TMA, amino acid bioconversion, and drug resistance.

We further investigated the hub genes related to glycan biosynthesis in our cohort fecal DNA. We firstly identified 15 orthologs as target hub genes based on glycan biosynthesis pathway information from KEGG and Genecards databases^5^ ^,6^ and the 15 hub genes are shown in Supplementary Table 9. Then we looked for the 15 hub genes through our cohort metagenomic results and found that five of them were detected in our cohort fecal DNA samples. Among the five hub genes, four of them are involved in *N*-glycan biosynthesis, and the last one is involved in *O*-glycan biosynthesis. As shown in Supplementary Figure 2, three of the five hub genes had higher abundances in the HBP group, while the other two (including the *O*-glycan biosynthesis-related ortholog) were lower in the HBP group, but none of them obtained significantly abundant difference between the two groups.

### Untargeted Metabolomics Analysis Identified a Series of Significantly Changed Metabolites in Fecal and Plasma Samples

We carried out untargeted LC-MS analysis and at the same time examined metagenomics, in order to evaluate the actual metabolic changes in fecal and plasma samples of our cohort. All detected compounds with VIP > 1 were assigned to metabolites and underwent further statistical analysis. As shown in Supplementary Tables 10, 11, there were a total of 353 types of compounds in fecal samples, and 217 types of compounds in plasma samples were assigned to metabolites. The assigned metabolites with *p*-value < 0.05 were found to be significantly different between healthy control and HBP patients. According to literature (Liu et al., 2018), the LC-MS condition for glycan detection is different from our method, and we did not expect to see metagenomic evidence of glycan changes when we carried out LC-MS, and samples were used up, so our LC-MS did not detect glycan compounds.

**FIGURE 3:**
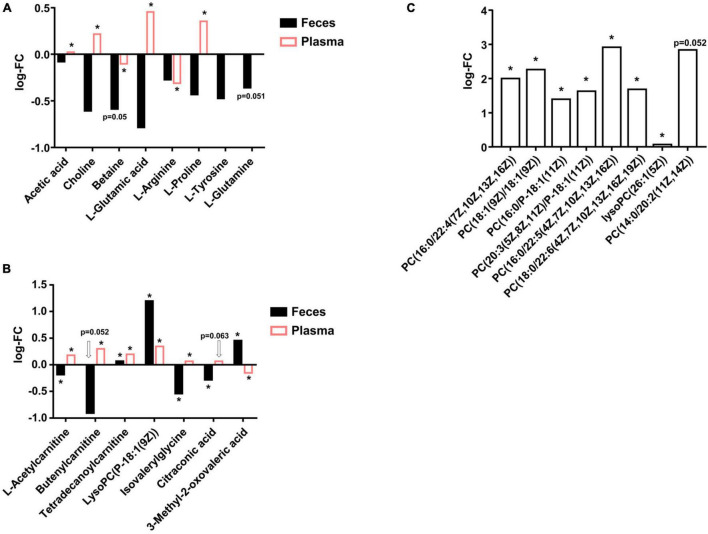
Liquid chromatography–mass spectrometry (LC-MS) detected metabolites differences in fecal and plasma samples between healthy control and primary hypertension patients. **(A)** Actual changes of short-chain fatty acid (SCFA), trimethylamine (TMA) precursors, and amino acids in fecal and plasma samples predicted by metagenomic analysis. **(B)** Metabolites were significantly changed in both fecal and plasma samples. **(C)** Phosphocholines were significantly higher in hypertension (HBP) fecal samples. FC, fold change of HBP group to Ctrl group. PC, phosphocholines. *p*-values were evaluated by two-tailed Welch’s *t*-test based on compounds’ peak intensities. *p < 0.05.

We firstly evaluated the changes of SCFA, TMA precursors, and some amino acids according to metagenomic results stated in Section “Abundances of Metagenomic Functional Categories Related to Glycan, Short-Chain Fatty Acid, Trimethylamine Precursor, Amino Acid Metabolism, and Drug Resistance Greatly Changed in Hypertension Patients.” As shown in Figure 3A, we found that, for SCFA members, only acetic acid had VIP > 1, and other SCFA members were not assigned to metabolites and underwent statistical analysis. LC-MS result suggested HBP fecal samples had lower acetic acid than healthy control, while HBP plasma samples had higher acetic acid, and such result is in accordance with the increased SCFA transporter ortholog in HBP fecal DNA. For TMA precursors, only choline and betaine had VIP > 1, and both of them were lower in HBP feces (while choline was higher in HBP plasma), and such results are also in accordance with metagenomic results. For metagenomic result suggested amino acid changes, glutamate (glutamic acid), arginine, proline, tyrosine, and glutamine had VIP > 1 in fecal samples; and all of them were lower in the HBP group, while only the first three amino acids were assigned in plasma samples. Moreover, only fecal glutamate and glutamine changes are in accordance with metagenomic ortholog changes.

Then we evaluated the compounds that were significantly changed in both fecal and plasma samples, and we identified seven metabolites, as shown in Figure 3B. The first three of the seven metabolites were derivatives of carnitine; the fourth is a derivative of phosphocholines (PC); the fifth is a derivative of glycine; and the last two are organic acids. These metabolites were mostly lower in HBP fecal samples but higher in HBP plasma, suggesting the enhanced intestinal epithelium transport functions and/or biosynthesis functions in HBP patients.

There were also a series of compounds only assigned in either fecal samples or plasma samples and were shown to be significantly different between healthy control and HBP patients. For instance, as shown in Figure 3C, there were eight kinds of PC significantly higher in HBP fecal samples, while there were 21 kinds of LysoPC significantly higher in HBP plasma samples (Supplementary Table 11). Such results suggested ability changes of some bioconversion networks linking intestinal system to whole body metabolism.

### Metagenomic Analysis on Macrophage-Specific Adenosine Monophosphate-Activated Protein Kinase-Knockout Hypertension Mice

After we identified the gut microbiome characteristics in primary hypertension patients with antihypertension medications, we attempted to uncover the potential mechanisms that caused such characteristics, and more specifically, which part of the gut microbiota characteristics were affected by such mechanisms. Meta-analysis on patients’ medications suggested macrophage-AMPK might mediate gut microbiota changes in our investigated patients (see Supplementary Material and Supplementary Table 2 for details). To test the potential influences of macrophage-AMPK mechanism on hypertension gut microbiota, we carried out AngII-induced hypertension model on macrophage-specific AMPK-knockout mice. The generation of macrophage-specific AMPK-knockout mice was as described in our previous publication (Zheng et al., 2019). And AngII significantly increased BP in wild-type (AW) and AMPK-knockout (AK) mice, as described in our recent article (Zheng et al., 2021). Fecal samples of wild-type and AMPK-knockout mice were collected before AngII stimulation (–A) and after AngII stimulation for 7 days (–B) and then underwent DNA extraction and next-generation sequencing on metagenomics. Sequencing by the Illumina HiSeq platform on 20 fecal DNA samples generated a total of 233.8 Gb of 150-bp paired-end reads, with an average of 11.7 Gb per sample. The raw data were further filtered and assembled to obtain Scaftigs and Unigenes for bioinformatics analysis, following standard protocol as processing individuals’ data. The sequencing generated data, and final Scaftigs information is summarized in Supplementary Table 12; and abundances of annotated taxonomies, KEGG orthologs, and KEGG pathways are summarized in Supplementary Tables 13–15.

**FIGURE 4:**
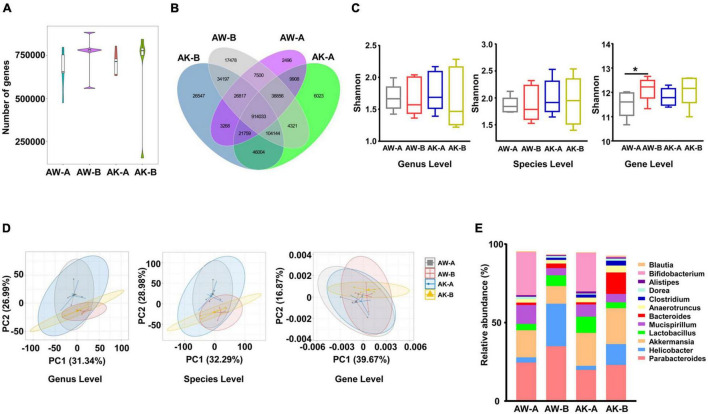
Microbial composition changes among wild-type and adenosine monophosphate-activated protein kinase (AMPK)-knockout mice before and after angiotensin II treatment. **(A)** Gene count comparison among the four groups. **(B)** Venn comparison of the shared gene numbers and unique gene numbers among the four groups. **(C)** α-Diversity analysis (Shannon index) based on genus, species, and ortholog profile in the four groups. **(D)** Principal component analysis (PCA) of β-diversity analysis based on genus, species, and ortholog profile among the four groups. **(E)** The relative abundances of the top 12 highest abundant genera among the four groups. AW-A, wild-type mice (AMPKα1^fl/fl^/WT, already received tamoxifen injection), which did not receive angiotensin II treatment. AW-B, wild-type mice received angiotensin II stimulation for 7 days. AK-A, AMPK-knockout mice (AMPKα1^fl/fl^/Csf1r-MerCre, already received tamoxifen injection), which did not receive angiotensin II treatment. AK-B, AMPK-knockout mice received angiotensin II stimulation for 7 days. Gene level, i.e., ortholog level. *p < 0.05 by one-tailed Mann–Whitney *U*-test.

After getting the clean sequencing data of mouse metagenomics, we also evaluated the general changes of gene content and bacterial composition. We found that the gene number was increased in both wild-type and AMPK-knockout mice after AngII stimulation (which was in contrast to the changes in human cohort), while without statistical significance (Figure 4A). And over 80% of the gene categories were shared by any two groups of mice either with different genotypes or at different time points (Figure 4B). Moreover, similar to that of the human cohort, the α-diversity calculated by Shannon index (Figure 4C) and β-diversity evaluated by PCA (Figure 4D) showed little difference between wild-type and AMPK-knockout mice before and after AngII stimulation at genus and species levels, while α-diversity was significantly higher in wild-type mice after AngII stimulation at ortholog level (gene level). Besides, α-diversity evaluated by Simpson and Evenness indexes showed similar changes as by Shannon index (Supplementary Figure 3). These results suggested that neither hypertension condition nor AMPK-knockout caused much changes on mouse gut microbiota genetic and taxonomic composition. Nevertheless, abundant changes were obvious in some genera and species after AngII stimulation and AMPK knockout. As showed in Figure 4E, among the top 12 highest abundant genera, *Bifidobacterium* was significantly reduced in both genotypes of mice after AngII stimulation, while *Alistipes* was significantly reduced in wild-type mice after AngII stimulation, and *Bacteroides* and *Helicobacter* were significantly increased in wild-type mice after AngII stimulation.

### Glycan Biosynthesis by Gut Microbiota Was Potentially Enhanced by Macrophage-Adenosine Monophosphate-Activated Protein Kinase Under Hypertension

We then compared the abundant changing trend of bacterial taxonomies, orthologs, and pathways from basal level to hypertension level in human and mouse metagenomics, so as to identify which part of the significantly changed metagenomic components may be affected by macrophage-AMPK in our investigated patients. The basic principle for comparison is that, if a significantly changed metagenomic characteristic in human cohort (either increase or decrease in HBP patients) shows the same changing trend in wild-type mice after AngII stimulation, while showing reduced (less than in wild-type mice) or reversed changing trend in AMPK-KO mice after AngII stimulation, then such characteristic should potentially be affected by macrophage-AMPK mechanism.

**FIGURE 5:**
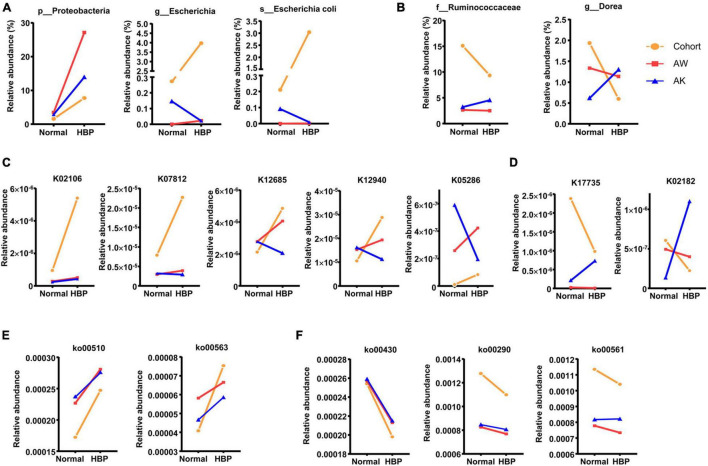
Significantly different gut microbiome components between healthy control and primary hypertension patients that are potentially affected by macrophage–adenosine monophosphate-activated protein kinase (AMPK) mechanism. **(A)** Significantly different bacterial strains that are potentially promoted after macrophage-AMPK activation, and their relative abundant changes from normal blood pressure groups to hypertension groups. **(B)** Significantly different bacterial strains that are potentially inhibited after macrophage-AMPK activation, and their relative abundant changes from normal blood pressure to hypertension condition. **(C)** Significantly different orthologs that are potentially promoted after macrophage-AMPK activation, and their relative abundant changes from normal blood pressure to hypertension condition. **(D)** Significantly different orthologs that are potentially inhibited after macrophage-AMPK activation, and their relative abundant changes from normal blood pressure groups to hypertension groups. **(E)** Significantly different pathways that are potentially promoted after macrophage-AMPK activation, and their relative abundant changes from normal blood pressure to hypertension condition. **(F)** Significantly different pathways that are potentially inhibited after macrophage-AMPK activation, and their relative abundant changes from normal blood pressure groups to hypertension groups. In each graph, the orange line shows change trend from healthy control group (Normal) to primary hypertension patient group with antihypertension medications (HBP), the red line shows change trend in wild-type mice from basal level (Normal) to AngII-induced hypertension level (HBP), and the blue line shows change trend in AMPK-knockout mice from basal level (Normal) to AngII-induced hypertension level (HBP). The meanings of AW and AK are the same as in Figure 4.

Based on this principle, we found that the HBP patients enriched *Escherichia* and *E. coli*, and their phylum Proteobacteria, which may be upregulated by macrophage-AMPK mechanism, as their abundances were less increased or decreased after AngII stimulation in AMPK-KO mice (Figure 5A). Reversely, healthy individuals enriched *Dorea* and *Ruminococcaceae* may be inhibited by macrophage-AMPK mechanism in HBP patients (Figure 5B). As for significantly changed KEGG orthologs, the SCFA transporter ortholog K02106, the TMAO reductase ortholog K07812, two orthologs for amino acid metabolism (K12685 and K12940), and the ortholog for phosphatidylinositol glycan biosynthesis (K05286) should be upregulated by macrophage-AMPK (Figure 5C), while two orthologs for carnitine metabolism (K17735 and K02182) should be inhibited by macrophage-AMPK (Figure 5D). And among significantly changed KEGG pathways, two pathways related to glycan biosynthesis (ko00510 and ko00563) should be upregulated by macrophage-AMPK (Figure 5E), while pathways for taurine metabolism (ko00430), leucine/valine/isoleucine biosynthesis (ko00290), and glycerolipid metabolism (ko00561) should be inhibited by macrophage-AMPK (Figure 5F). These results suggested that, in primary hypertension patients with antihypertension medications, the AMPK activation in macrophage could mainly promote gut microbiota biosynthesis of glycan, while such mechanism could also regulate amino acid metabolism and some other functions of gut microbiota.

## Discussion

The animal models and human cohort studies have proved the causality of gut microbiota to hypertension (Karbach et al., 2016; Li et al., 2017) and identified a series of gut microbiota strains that had significantly abundant changes in pretreatment HBP patients, such as *Klebsiella*, were enriched in the HBP group (Li et al., 2017), while *Faecalibacterium* and *Dorea* were enriched in healthy controls (Li et al., 2017, 2019). Some of the gut microbiota-derived metabolites were proved to be mediators for gut microbiota participating in hypertension development. For instance, SCFA is mainly believed to reduce BP, while TMA is believed to promote hypertension (Jaworska et al., 2020). Still, among hypertension patients receiving antihypertension medications, the metagenomic changes are not clear, let alone the mechanisms that affect gut microbiota changes.

In this study, we investigated the metagenomic characteristics in hypertension patients who were taking antihypertension drugs, and we identified a series of gut microbiota strains that showed significantly abundant changes. Some of the changes were in accordance with previous studies on pretreated hypertension patients in China, such as the increase of *Klebsiella*, *Eubacterium*, and *F. prausnitzii* (Yan et al., 2017; Kim et al., 2018) and decrease of *Faecalibacterium* and *Dorea*; and these bacterial changes could be common signs of hypertension. And other significant changed bacteria, for instance, the increase of *B. fragilis* in our investigated patients, should be unique under medication treatment.

As for the potential gut microbiota functional changes in our investigated patients, some of the SCFA-generating bacteria were enriched, while some others were decreased, and similar conditions were found in TMA-generating bacteria changes. Correspondingly, some KEGG categories related to SCFA utilization and TMA precursor metabolism were either increased or decreased in HBP patients. Untargeted LC-MS proved the decrease of acetic acid, choline, and betaine in HBP fecal samples, and it is in accordance with metagenomic results, while amino acid changes did not exactly match metagenomic results, suggesting more complex metabolic network on amino acid utilization. Except for SCFA and TMA, we found that some metagenomic characteristics around glycan biosynthesis were significantly increased in hypertension patients. Members of *Bacteroidetes* are the main producers of complex glycans (Martens et al., 2009), and *B. fragilis* as one of the glycan producers (Coyne et al., 2008) was enriched in the HBP group. Correspondingly, there were four glycan biosynthesis-related pathways greatly enriched in the HBP group, and two of them were for *N*-glycan biosynthesis. And *N*-glycan precursor biosynthesis module was also greatly increased in the HBP group. Such enhancing ability of generating glycan should have unique metagenomic changes under antihypertension medications.

Meta-analysis on the medications’ common mechanism and immuno–gut microbiota interactions suggested AMPK pathway and macrophage as the regulators of gut microbiota in HBP patients taking antihypertension medication, and we verified such hypothesis by using macrophage-specific AMPK-knockout mouse hypertension model. Since AngII could directly active AMPK (Day et al., 2011), the AngII stimulation could mimic medication stimulation effects on macrophage under hypertension. The metagenomic change intersection in HBP patients and wild-type hypertension mice was further filtered by metagenomic changes in AMPK-knockout hypertension mice, as described in the section “Results.” By such strategy, we found that among significantly changed bacteria in HBP patients, *Dorea* could be inhibited by macrophage-AMPK mechanism in HBP patients. Among significantly changed KEGG categories in HBP patients, macrophage-AMPK mechanism potentially activated one SCFA transporter ortholog, one TMAO reductase ortholog, and two glycan biosynthesis pathways, while it inhibited two carnitine-metabolic orthologs. Meanwhile, some metagenomic functions around amino acid metabolisms were also potentially affected by macrophage-AMPK. Thus, the macrophage-AMPK mechanism could affect multiple aspects of gut microbiota functions under antihypertension medication treatment, and medication influences on macrophage-AMPK should be considered during hypertension treatment.

Glycans, especially *N*-glycans, affect their decorated protein folding, stability, and functions (Cao et al., 2018). They are also a part of extracellular matrix and are crucial for intercellular communication (Bojar et al., 2021). Glycans also mediate gut microbiota colonization (Rogowski et al., 2015). Our results suggested that the antihypertension medications might enhance intracellular interactions between gut bacteria and intestinal cells by enhancing glycan biosynthesis of gut microbiota, and such metagenomic changes were potentially regulated through macrophage-AMPK (which was reasonable considering the regulation effects of macrophage on gut microbiota). Moreover, microbial glycans were recently reported to mimic host glycans, so as to regulate host immunity (Bojar et al., 2021), while some kind of host IgG *N*-glycans were significantly increased in hypertension patients (Liu et al., 2018) and potentially promoted hypertension through regulating vascular smooth muscle cell contraction and hypertrophy (Song et al., 2021), so the gut microbiota-derived glycans might have direct effects on host immune activity and hypertension development. Because of the different LC-MS conditions for glycan detection and sample used, we did not detect the actual glycan contents in fecal and plasma samples. Further studies are needed to verify the actual glycan changes in HBP fecal samples and to verify the possibility of gut microbiota-derived glycans mimicking host glycans’ role on immune system.

There are also some other limitations in our study. First, because of the small sample size, the α-diversity in HBP patients was reduced, but not very significant, while the β-diversity showed little difference (Figure 1 and Supplementary Figure 1). According to reference (Casals-Pascual et al., 2020), if the sample size is enlarged to above 50 people in each group, then the diversity changes should be more obvious. Second, our study did not take into account HBP patients without medications. Such limitation is because of the difficulties of recruiting newly diagnosed HBP patients without medication treatment and fecal sample collection, and the uncertainty of which part of our observed microbiome changes was mainly caused by the medication. Besides, the average age, FBG, and TG were higher in HBP patients, while HDL was lower in HBP patients (Table 1), and these factors might affect microbiome differences between healthy control and HBP patients. So we evaluated the correlation of these factors with some obviously changed gut microbiome components separately, and we found that only a few of components were significantly correlated with the factors (Supplementary Table 16). Among these significant correlations, age and FBG showed none correlation with glycan biosynthesis-related bacteria or orthologs, while TG only positively correlated with one glycan-generating bacteria (*B. vulgatus*), and HDL only negatively correlated with the same bacteria, so these factors should have little influence on at least glycan-biosynthesis bacteria and functions of gut microbiome in HBP patients. Still, there was no body mass index (BMI) information in the HBP group, making us unable to analyze the correlation of BMI and the identified metagenomic changes. But according to meta-analysis on obesity adults’ gut microbiota (Castaner et al., 2018), the obesity adults had enriched *Blautia hydrogenotrophica*, *Coprococcus catus*, *Eubacterium ventriosum*, *Ruminococcus bromii*, and *Ruminococcus obeum*; while lean adults had higher abundance of *Bacteroides faecichinchillae*, *Bacteroides thetaiotaomicron*, *Clostridium bolteae*, and *Flavonifractor plautii*. These bacteria are not the significantly different bacteria in our study, so we believe that BMI/obesity is not the major cause of the identified metagenomic changes in this study. Future works are needed to verify the metagenomic changes found in this study in a larger cohort and to identify the actual metabolite changes and their physiological functions.

**FIGURE 6:**
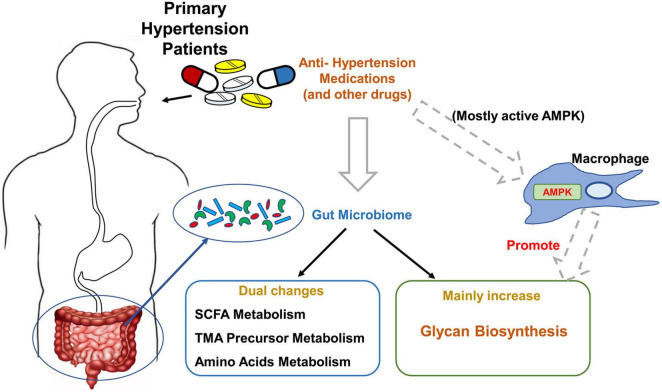
Summary of the major findings of this study. The major changes of gut microbiome in primary hypertension patients taking antihypertension medications, and potential regulating mechanism by macrophage–adenosine monophosphate-activated protein kinase (AMPK) are summarized in this schema.

In summary, our study identified the metagenomic changes around glycan, SCFA, TMA, and amino acid metabolism in primary hypertension patients with antihypertension medications, especially enhanced glycan biosynthesis ability; verified several of SCFA, TMA precursors, and amino acid changes in fecal and plasma samples by LC-MS; and uncovered the potential influence of macrophage-AMPK on these metagenomic changes (Figure 6). Our results suggest gut microbiota-derived glycan as a potentially new target for hypertension study, and macrophage-AMPK pathway activation should be considered during antihypertension treatment.

## Data Availability Statement

The datasets presented in this study can be found in online repositories. The names of the repository/repositories and accession number(s) can be found below: https://www.ncbi.nlm.nih.gov/, PRJNA685581.

## Ethics Statement

The studies involving human participants were reviewed and approved by the Clinical Research Ethics Committee of Anzhen Hospital. The patients/participants provided their written informed consent to participate in this study. The animal study was reviewed and approved by the Ethics Committee of Anzhen Hospital.

## Author Contributions

SZ, BZ, and JD conceived the project and wrote the manuscript. BZ and JD supervised the project and revised the manuscript. SZ, XL, and JR recruited patients and healthy volunteers and collected related information. SZ and YuL collected cohort fecal samples. SZ, CP, YaL, XL, TL, and XZ carried out animal model studies. SZ analyzed the data. All the authors contributed to the article and approved the submitted version.

## Conflict of Interest

The authors declare that the research was conducted in the absence of any commercial or financial relationships that could be construed as a potential conflict of interest. The reviewer JW declared a shared affiliation with one of the author, BZ, to the handling editor at time of review.

## Publisher’s Note

All claims expressed in this article are solely those of the authors and do not necessarily represent those of their affiliated organizations, or those of the publisher, the editors and the reviewers. Any product that may be evaluated in this article, or claim that may be made by its manufacturer, is not guaranteed or endorsed by the publisher.

## Footnotes

1
https://www.ncbi.nlm.nih.gov/sra/PRJNA685581

2
http://www.metaboanalyst.ca/MetaboAnalyst/

3
http://www.hmdb.ca/

4
https://metlin.scripps.edu/

5
www.kegg.jp

6
www.genecards.org

## Abbreviations

AMPK: adenosine 5′-monophosphate-activated protein kinase
Ang II: angiotensin II
BMI: body mass index
BP: blood pressure
Ctrl: control group
DBP: diastolic blood pressure
FBG: fasting blood glucose
HBP, high blood pressure group: hypertension group
HDL: high-density lipoprotein
KEGG: Kyoto Encyclopedia of Genes and Genomes
KO: KEGG ortholog group
LC-MS: liquid chromatography–MS
LDL: low-density lipoprotein
LPS: lipopolysaccharide
PC: phosphocholines
PCA: principal component analysis
SBP: systolic blood pressure
SCFA: short-chain fatty acid
TC: total cholesterol
TG: triglyceride
TMA: trimethylamine
TMAO: trimethylamine *N*-oxide.
