# Supplementary material for: Glycan Biosynthesis Ability of Gut Microbiota Increased in Primary Hypertension Patients Taking Antihypertension Medications and Potentially Promoted by Macrophage-Adenosine Monophosphate-Activated Protein Kinase
Source: Front Microbiol. 2021 Nov 4;12:719599. doi: 10.3389/fmicb.2021.719599 (PMC8600050; doi:10.3389/fmicb.2021.719599)
Supplement: Supplementary file 2 [file Data_Sheet_1.pdf]

Supplementary Material

1 Supplementary Data Information

This file includes:

Supplementary information of meta-analysis on medications' effects on AMPK pathway.

Supplementary Figure legend 1 to 3

Supplementary Table 2.

Supplementary table legend 1 to 16.

Supplementary references

2 Supplementary Information of Meta-analysis on Medications' Effects on AMPK Pathway

From literature study, we found that half of the drugs (36 in 72) in the medication history of our investigated patients were reported to regulate AMP-activated protein kinase (AMPK) pathway in different tissues (Supplementary Table 2), and 34 of the 36 drugs were reported to active AMPK pathway. Moreover, 26 of the 30 investigated patients took at least one kind of the 34 AMPK-activated drugs during the investigation and/ or right before coming to our hospital, and the rest 4 patients took drugs having dual-regulation effects on AMPK (Supplementary Table 1). On the other side, hypertension could activate macrophage to induce systemic inflammation and cause organs damage (De Ciuceis et al., 2005； Tieu et al., 2009), while macrophage along with other immune cells could regulate gut microbiota colonization and composition (Earley et al., 2018; Mao et al., 2018), and AMPK pathway is essential for regulating macrophage phenotype and functions (Sag et al., 2008). Based on these cognitions, we hypothesized that, the macrophage-AMPK mechanism might be the common regulation mechanism of anti-hypertension medications to mediate gut microbiota changes in our investigated patients.

3 Supplementary Figure Legends

Supplementary Figure 1. α-diversity analysis by Simpson and Evenness index in human cohort

α-diversity analysis by Simpson and Evenness index based on genus (A), species (B), and orthologs (C) profile. Ctrl, healthy control group. HBP, primary hypertension patient group who took anti-hypertension medications before testing metagenomics. Gene level, i.e., ortholog level.

Supplementary Figure 2. Abundant differences of several hub genes related to glycan biosynthesis

The abundances of 5 glycan-biosynthesis related hub genes, which were identified in cohort fecal DNA, were shown here. Ctrl, healthy control group. HBP, hypertension patient group. P=0.054 was evaluated by two-tailed Mann-Whitney U-test.

Supplementary Figure 3. α-diversity analysis by Simpson and Evenness index in experimental mice

α-diversity analysis by Simpson and Evenness index based on genus (A), species (B), and orthologs (C) profile. AW-A, wild-type mice (AMPKα$1^{fl/fl}$/WT, already received tamoxifen injection), which did not receive angiotensin II treatment. AW-B, wild-type mice received angiotensin II stimulation for 7 days. AK-A, AMPK-knockout mice (AMPKα$1^{fl/fl}$/Csf1r-MerCre, already received tamoxifen injection), which did not receive angiotensin II treatment. AK-B, AMPK-knockout mice received angiotensin II stimulation for 7 days. Gene level, i.e., ortholog level. *, $p < 0.05$ by one-tailed Mann-Whitney U-test. P=0.06 was also evaluated by one-tailed Mann-Whitney U-test.

4 Supplementary Tables

Supplementary Table 1. Basic information of study cohort

The basic physiological index, medical history and medication history of the 8 healthy people and 30 primary hypertension patients are shown in the table. For primer hypertension patients, the BMI was not recorded in the medical history system. Blood biochemical index was labeled red or blue if the value was higher or lower than the normal range. Drugs with red color are reported to activate AMPK pathway, while drugs with blue color are reported to inhibit AMPK pathway, and drugs with green color are reported to be able to both activate and inhibit AMPK pathway. Patients labeled with yellow color are taking AMPK-activated drugs during the current medical treatment and before fecal sample collection, or took AMPK-activated drugs before the current medical treatment, or both. And patients labeled with orange color are taking AMPK-dual-function drugs without AMPK-activated drugs during the current medical treatment and before fecal sample collection, or took such medication before the current treatment, or both. Control is defined as SBP ≤125 mmHg and DBP ≤80 mmHg for untreated subjects. Hypertension is defined as current SBP ≥130 mmHg or DBP ≥90 mmHg patients with or without antihypertension treatments. Ctrl, healthy control group; HBP, hypertension patient group; SBP, systolic blood pressure; DBP, diastolic blood pressure; BMI, body mass index; FBG, fasting blood glucose; TC, total cholesterol; TG, triglyceride; HDL, high density lipoprotein; LDL, low density lipoprotein. For HBP patients, their blood pressure control effects were based on the BP values when discharged from hospital, according to the standard in "2018 Chinese Guidelines for Prevention and Treatment of Hypertension".

Supplementary Table 2. Regulation effects of drugs in the cohort medication history on AMPK pathway

Drug Code	Drug Name	Indication Symptom	Regulation effect on AMPK pathway, and tissue/cell type	Reference
#01	Micardis, Telmisartan	Hypertension	Activate<br><br>Vascular smooth muscle cells	(Hwang and Cho, 2020)
#02	Losartan Potassium	Hypertension	Activate<br><br>H9c2 rat cardiomyocytes	(Hernandez et al., 2014)
#03	Hyzaar; Losartan potassium and hydrochlorothiazide tablets	Hypertension	Activate<br><br>H9c2 rat cardiomyocytes	(Hernandez et al., 2014)
#04	Salcuba trivalsartan sodium	Hypertension	No report	/
#05	Plendil; Felodipine	Hypertension	No report	/
#06	Benidipine	Hypertension	Activate<br><br>Rat kidney	(Ishizaka et al., 2010)
#07	Benidipine hydrochloride	Hypertension	Activate<br><br>Rat kidney	(Ishizaka et al., 2010)
#08	Lercanidipine	Hypertension	No report	/
#09	Lercanidipine hydrochloride	Hypertension	No report	/

#10	Levamlodipine	Hypertension	No report	/
#11	Norvasc, Amlodipine besylate tablets	Hypertension	No report	/
#12	Allisartan isoproxil	Hypertension	No report	/
#13	Olmesartan	Hypertension	Not regulate<br><br>Mouse glomerulus	(Gu et al., 2016)
#14	Candesartan cilexetil	Hypertension	Activate<br><br>Rat hypothalamic tissue	(Marques et al., 2012)
#15	Adalat, Nifedipine	Hypertension	Both activate and inhibit<br><br>Activate: vascular smooth muscle cells<br><br>Inhibit: Rat kidney	Active: (Sung and Choi, 2012)<br><br>Inhibit: (Lin et al., 2020)
#16	Nifedipine sustained-release tablets	Hypertension	Both activate and inhibit<br><br>Activate: vascular smooth muscle cells<br><br>Inhibit: Rat kidney	Active: (Sung and Choi, 2012)<br><br>Inhibit: (Lin et al., 2020)
#17	Diovan, Valsartan capsules	Hypertension	Activate<br><br>THP-1 cells, monocyte-like cell line	(Ha et al., 2014)
#18	Co-Diovan, Valsartan and	Hypertension	Activate	(Ha et al., 2014)

	hydrochlorothiazide tablets		THP-1 cells, monocyte-like cell line	
#19	Valsartan amlodipine tablets	Hypertension	Activate<br><br>THP-1 cells, monocyte-like cell line	(Ha et al., 2014)
#20	Irbesartan	Hypertension	Activate<br><br>Human and mouse hepatocytes	(He et al., 2019)
#21	Aprovel, Irbesartan	Hypertension	Activate<br><br>Human and mouse hepatocytes	(He et al., 2019)
#22	Irbesartan and hydrochlorothiazide tablets	Hypertension	Activate<br><br>Human and mouse hepatocytes	(He et al., 2019)
#23	Terazosin	Hypertension	No report	/
#24	Terazosin hydrochloride tablets	Hypertension	No report	/
#25	Furosemide	Hypertension	Inhibit<br><br>Human liver (HepG2) and primary kidney (HRCE) cells	(Knake et al., 2014)
#26	Acertil, Perindopril tablets	Hypertension	No report	/
#27	Natrilix tablets, Indapamide	Hypertension	No report	/

#28	Spironolactone	Hypertension	Activate<br><br>Dog ventricular tissues	(Li et al., 2019)
#29	Reserpine，Dihydralazine，Hydrochlorothiazide	Hypertension	No report	/
#30	Compound hypotensive tTablets	Hypertension	No report	/
#31	Herbesser, Diltiazem hydrochloride	Hypertension	Inhibit<br><br>Neuronal differentiated PC12 cells	(Lee et al., 2016)
#32	Bisoprolol hemifumarate	Hypertension	No report	/
#33	Labetalol	Hypertension	No report	/
#34	Arotinolol	Hypertension	No report	/
#35	Betaloc, Metoprolol	Hypertension	Activate<br><br>Dog cardiac tissue	(Sun et al., 2017)
#36	Concor, Bisoorolol	Hypertension	No report	/
#37	Fosinopril	Hypertension	No report	/
#38	Enalapril	Hypertension	Activate<br><br>Mouse cardiac tissue	(Suarez-Martinez et al., 2014)
#39	Benazepril	Hypertension	No report	/
#40	Indapamide tablets	Hypertension	No report	/

#41	Carvedilol	Hypertension	Activate<br><br>Cardiomyocytes	(Hu et al., 2019)
#42	Lacidipine	Hypertension	No report	/
#43	Verapamil, Verapamil hydrochloride	Hypertension and arrhythmia	No report	/
#44	Potassium chloride sustained-release tablets	Hypertension and arrhythmia	No report	/
#45	Basalin, Glargine	Diabetes	Activate<br><br>Rat gastrocnemius muscle	(Hong et al., 2018)
#46	Glucobay, Acarbose	Diabetes	Activate<br><br>Rat duodenal mucosal	(Duca et al., 2015)
#47	Metformin	Diabetes	Activate<br><br>Rat duodenal mucosal	(Duca et al., 2015)
#48	Glucophage, Metformin hydrochloride tablets	Diabetes	Activate<br><br>Rat duodenal mucosal	(Duca et al., 2015)
#49	Sitagliptin phosphate	Diabetes	Activate<br><br>Mouse endothelial progenitor cells	(Dai et al., 2018)
#50	Pitavastatin, Livalo	Hyperlipidemia	Activate<br><br>L6 skeletal muscle cells	(Ohira et al., 2012)

#51	Pitastatin calcium tablets	Hyperlipidemia	Activate<br><br>L6 skeletal muscle cells	(Ohira et al., 2012)
#52	Atorvastatin, Lipitor	Hyperlipidemia	Activate<br><br>L6 skeletal muscle cells	(Ohira et al., 2012)
#53	Rosuvastatin	Hyperlipidemia	Activate<br><br>SH-SY5Y cells, neuroblastic cell cluster	(Kang et al., 2017)
#54	Xuezhikang Capsule	Hyperlipidemia	No report	/
#55	Lipanthyl, Fenofibrate	Hyperlipidemia	Activate<br><br>Gastric cancer cells	(Chen et al., 2020)
#56	Ezetimibe	Hyperlipidemia	Activate<br><br>Mice adipocyte tissue	(Lee et al., 2020)
#57	Ethyl polyenoate soft capsules	Hyperlipidemia	No report	/
#58	Aspirin	Coagulation	Activate<br><br>Hepatocellular carcinoma cells	(Huang et al., 2018)
#59	Plavix, Clopidogrel hydrogen sulfate	Coagulation	Inhibit<br><br>HL-60 cells, leukemic cell line	(Yang et al., 2016)
#60	Dabigatran	Coagulation	No report	/

#61	Bayaspirin	Coagulation	Activate<br><br>Human hepatocellular carcinoma cell	(Huang et al., 2018)
#62	Warfarin	Coagulation	No report	/
#63	Imdur, Isosorbide mononitrate sustained-release tablets	Coronary heart disease	No report	/
#64	Zhenyuan capsule	Coronary heart disease	No report	/
#65	Clarityne, Loratadine	Allergic rhinitis	Not regulate<br><br>MC3T3-E1 cells, osteoblastic cell line	(Sun et al., 2019)
#66	Febuxostat	Gout	Activate<br><br>Renal tubular cells	(Kim et al., 2020)
#67	Pantoprazole sodium	Peptic ulcer	No report	/
#68	Rabeprazole sodium	Peptic ulcer	No report	/
#69	Zoloft, Sertraline hydrochloride	Depression	Activate<br><br>Human umbilical vein endothelial cells	(Hwang et al., 2020)
#70	Zopiclone	Insomnia	No report	/
#71	Trastal, Piribedil tablets	Parkinson	No report	/
#72	Madopar, Levodopa and benserazide	Parkinson	No report	/

Drugs which were reported to activate AMPK are labeled red, and drugs which were reported to inhibit AMPK are labeled blue. Two drugs were reported to both activate and inhibit AMPK pathway in different tissues, and they are labeled green.

Supplementary Table 3. Sequencing data production of 38 fecal DNA samples of study cohort

Raw data is the original data after sequencing. Clean data is the effective data after filtering the raw data. Scaftigs are the continuous sequences within scaffolds, which were generated from clean data by assembly analysis using SOAPdenovo software. Total length is the length of all Scaftigs in each sample. N50 length is the length of a medial Scaftig sequence when its length plus all the lengths of sequences longer than it equals 50% of the total Scaftigs length. Ctrl, healthy control group. HBP, hypertension patient group.

Supplementary Table 4. Relative abundance of all identified bacterial taxonomies in study cohort fecal DNA

The abundance proportions (%) of all identified bacterial strains from phylum to species level in each sample are shown in the table.

Supplementary Table 5. Relative abundance of all annotated KEGG orthologs in study cohort fecal DNA

The abundance ratio of all annotated KEGG orthologs in each sample are shown in the table.

Supplementary Table 6. Significantly enriched KEGG orthologs in HBP group

The major part of significantly enriched KEGG orthologs and some of the nearly significant enriched orthologs in HBP group are shown in the table. The relative abundance of each ortholog is shown as

mean ± standard deviation. P value was evaluated by two-tailed Mann-Whitney test. Ctrl, healthy control group. HBP, hypertension patient group. Mean, average abundance. SD, standard deviation.

Supplementary Table 7. Relative abundance of all annotated KEGG pathways in study cohort fecal DNA

The abundance ratio of all annotated KEGG pathways in each sample are shown in the table.

Supplementary Table 8. Relative abundance of all annotated KEGG modules in study cohort fecal DNA

The abundance ratio of all annotated KEGG modules in each sample are shown in the table.

Supplementary Table 9. Hub genes related to glycan biosynthesis, and their abundances in study cohort fecal DNA

The major hub genes related to glycan biosynthesis were identified based on KEGG and Genecards databases, and their KEGG orthologs and KO ID are shown in the table. Only 5 of the hub genes were identified in the cohort fecal DNA, and their abundant differences between healthy control (Ctrl) and hypertension patients (HBP) were evaluated by two-tailed Mann-Whitney test. Mean, average abundance. SD, standard deviation.

Supplementary Table 10. Identified metabolites in study cohort fecal samples

Compounds in cohort fecal samples detected by LC-MS with VIP>1 were assigned to metabolites in HMDB, and then assigned to KEGG metabolomics pathways. The related information of these compounds is shown in the table. VIP, variable important in projection. FC, fold change of HBP group value to control group value. P value is evaluated by two-tailed Welch's t-test based on compounds peak intensities differences between control group and HBP group.

Supplementary Table 11. Identified metabolites in study cohort plasma samples

Compounds in cohort plasma samples were also detected and analysis as compounds in fecal samples, and results are shown in this table. The meanings of VIP, FC, and p value are the same as in Supplementary Table 10.

Supplementary Table 12. Sequencing data production of mice fecal samples

The meanings of Raw data, clean data, Scaftigs, total length, and N50 length are the same as in Supplementary Table S3. AW, AMPKα$1^{fl/fl}$/WT mice. AK, AMPKα$1^{fl/fl}$/Csf1r-MerCre mice. AW/AK-A, before Angiotensin II perfusion (after tamoxifen intraperitoneal for 5 days). AW/AK-B, after Angiotensin II perfusion for 7 days.

Supplementary Table 13. Relative abundance of all identified bacterial taxonomies in mice fecal DNA

The abundance proportions (%) of all identified bacterial strains from phylum to species level in each mouse fecal sample are shown in the table. The meanings of AW/AK-A/B are the same as in Supplementary Table 12.

Supplementary Table 14. Relative abundance of all annotated KEGG orthologs in mice fecal DNA

The abundance ratio of all annotated KEGG orthologs in each mouse sample are shown in the table. The meanings of AW/AK-A/B are the same as in Supplementary Table 12.

Supplementary Table 15. Relative abundance of all annotated KEGG pathways in mice fecal DNA

The abundance ratio of all annotated KEGG pathways in each mouse fecal sample are shown in the table. The meanings of AW/AK-A/B are the same as in Supplementary Table 12.

Supplementary Table 16. Spearman correlation test of physiological parameters with some gut microbiome components

The spearman correlation coefficient values (r) and statistical significance (p) are shown in the table. Listed gut microbiome components are greatly changed bacterial strains, orthologs, pathways, and module which are shown in Figure 2 and Figure 5. Components labeled in red color are increased in HBP group, and components labeled in blue color are decreased in HBP group. $p < 0.05$ is considered as statistically significant difference, and labeled yellow color and “*”.

5 Supplementary References

Chen, L., Peng, J., Wang, Y., Jiang, H., Wang, W., Dai, J., et al. (2020). Fenofibrate-induced mitochondrial dysfunction and metabolic reprogramming reversal: the anti-tumor effects in gastric carcinoma cells mediated by the PPAR pathway. *Am J Transl Res*. **12**, 428-446

Dai, X., Zeng, J., Yan, X., Lin, Q., Wang, K., Chen, J., et al. (2018). Sitagliptin-mediated preservation of endothelial progenitor cell function via augmenting autophagy enhances ischaemic angiogenesis in diabetes. *J Cell Mol Med*. **22**, 89-100. https://doi.org/10.1111/jcmm.13296

De Ciuceis, C., Amiri, F., Brassard, P., Endemann, D. H., Touyz, R. M., Schiffrin, E. L. (2005). Reduced vascular remodeling, endothelial dysfunction, and oxidative stress in resistance arteries of angiotensin II-infused macrophage colony-stimulating factor-deficient mice: evidence for a role in inflammation in angiotensin-induced vascular injury. *Arterioscler Thromb Vasc Biol*. **25**, 2106-2113. https://doi.org/10.1161/01.ATV.0000181743.28028.57

Duca, F. A., Cote, C. D., Rasmussen, B. A., Zadeh-Tahmasebi, M., Rutter, G. A., Filippi, B. M., et al. (2015). Metformin activates a duodenal Ampk-dependent pathway to lower hepatic glucose production in rats. *Nat Med*. **21**, 506-511. https://doi.org/10.1038/nm.3787

Earley, A. M., Graves, C. L., Shiau, C. E. (2018). Critical Role for a Subset of Intestinal Macrophages in Shaping Gut Microbiota in Adult Zebrafish. *Cell Rep*. **25**, 424-436. https://doi.org/10.1016/j.celrep.2018.09.025

Gu, J., Yang, M., Qi, N., Mei, S., Chen, J., Song, S., et al. (2016). Olmesartan Prevents Microalbuminuria in db/db Diabetic Mice Through Inhibition of Angiotensin II/p38/SIRT1-Induced Podocyte Apoptosis. *Kidney Blood Press Res*. **41**, 848-864. https://doi.org/10.1159/000452588

Ha, Y. M., Park, E. J., Kang, Y. J., Park, S. W., Kim, H. J., Chang, K. C. (2014). Valsartan independent of AT(1) receptor inhibits tissue factor, TLR-2 and -4 expression by regulation of Egr-1 through activation of AMPK in diabetic conditions. *J Cell Mol Med*. **18**, 2031-2043. https://doi.org/10.1111/jcmm.12354

He, J., Ding, J., Lai, Q., Wang, X., Li, A., Liu, S. (2019). Irbesartan Ameliorates Lipid Deposition by Enhancing Autophagy via PKC/AMPK/ULK1 Axis in Free Fatty Acid Induced Hepatocytes. *Front Physiol*. **10**, 681. https://doi.org/10.3389/fphys.2019.00681

Hernandez, J. S., Barreto-Torres, G., Kuznetsov, A. V., Khuchua, Z., Javadov, S. (2014). Crosstalk between AMPK activation and angiotensin II-induced hypertrophy in cardiomyocytes: the role of mitochondria. *J Cell Mol Med*. **18**, 709-720. https://doi.org/10.1111/jcmm.12220

Hong, O. K., Choi, Y. H., Kwon, H. S., Jeong, H. K., Son, J. W., Lee, S. S., et al. (2018). Long-term insulin treatment leads to a change in myosin heavy chain fiber distribution in OLETF rat skeletal muscle. *J Cell Biochem*. https://doi.org/10.1002/jcb.27571

Hu, H., Li, X., Ren, D., Tan, Y., Chen, J., Yang, L., et al. (2019). The cardioprotective effects of carvedilol on ischemia and reperfusion injury by AMPK signaling pathway. *Biomed Pharmacother*. **117**, 109106. https://doi.org/10.1016/j.biopha.2019.109106

Huang, Z., Fang, W., Liu, W., Wang, L., Liu, B., Liu, S., et al. (2018). Aspirin induces Beclin-1-dependent autophagy of human hepatocellular carcinoma cell. *Eur J Pharmacol*. **823**, 58-64. https://doi.org/10.1016/j.ejphar.2018.01.031

Hwang, H. Y., Shim, J. S., Kim, D., Kwon, H. J. (2020). Antidepressant drug sertraline modulates AMPK-MTOR signaling-mediated autophagy via targeting mitochondrial VDAC1 protein. *Autophagy*, 1-17. https://doi.org/10.1080/15548627.2020.1841953

Hwang, Y. J., Cho, D. H. (2020). Activation of AMPK/proteasome/MLCK degradation signaling axis by telmisartan inhibits VSMC contractility and vessel contraction. *Biochem Biophys Res Commun*. **524**, 853-860. https://doi.org/10.1016/j.bbrc.2020.02.007

Ishizaka, N., Hongo, M., Matsuzaki, G., Furuta, K., Saito, K., Sakurai, R., et al. (2010). Effects of the AT(1) receptor blocker losartan and the calcium channel blocker benidipine on the accumulation of lipids in the kidney of a rat model of metabolic syndrome. *Hypertens Res*. **33**, 263-268. https://doi.org/10.1038/hr.2009.224

Kang, S. Y., Lee, S. B., Kim, H. J., Kim, H. T., Yang, H. O., Jang, W. (2017). Autophagic modulation by rosuvastatin prevents rotenone-induced neurotoxicity in an in vitro model of Parkinson's disease. *Neurosci Lett*. **642**, 20-26. https://doi.org/10.1016/j.neulet.2017.01.063

Kim, H., Baek, C. H., Chang, J. W., Yang, W. S., Lee, S. K. (2020). Febuxostat, a novel inhibitor of xanthine oxidase, reduces ER stress through upregulation of SIRT1-AMPK-HO-1/thioredoxin expression. *Clin Exp Nephrol*. **24**, 205-215. https://doi.org/10.1007/s10157-019-01804-8

Knake, C., Stamp, L., Bahn, A. (2014). Molecular mechanism of an adverse drug-drug interaction of allopurinol and furosemide in gout treatment. *Biochem Biophys Res Commun*. **452**, 157-162. https://doi.org/10.1016/j.bbrc.2014.08.068

Lee, Y. J., Choi, S. Y., Yang, J. H. (2016). AMP-activated protein kinase is involved in perfluorohexanesulfonate-induced apoptosis of neuronal cells. *Chemosphere*. **149**, 1-7. https://doi.org/10.1016/j.chemosphere.2016.01.073

Lee, Y. S., Park, J. S., Lee, D. H., Han, J., Bae, S. H. (2020). Ezetimibe ameliorates lipid accumulation during adipogenesis by regulating the AMPK-mTORC1 pathway. *FASEB J*. **34**, 898-911. https://doi.org/10.1096/fj.201901569R

Li, H., Liu, B., Song, J., An, Z., Zeng, X., Li, J., et al. (2019). Characteristics of Gut Microbiota in Patients with Hypertension and/or Hyperlipidemia: A Cross-Sectional Study on Rural Residents in Xinxiang County, Henan Province. *Microorganisms*. **7**, 1-13. https://doi.org/10.3390/microorganisms7100399

Lin, Y. C., Wang, J. C., Wu, M. S., Lin, Y. F., Chen, C. R., Chen, C. Y., et al. (2020). Nifedipine Exacerbates Lipogenesis in the Kidney via KIM-1, CD36, and SREBP Upregulation: Implications from an Animal Model for Human Study. *Int J Mol Sci*. **21**. https://doi.org/10.3390/ijms21124359

Mao, K., Baptista, A. P., Tamoutounour, S., Zhuang, L., Bouladoux, N., Martins, A. J., et al. (2018). Innate and adaptive lymphocytes sequentially shape the gut microbiota and lipid metabolism. *Nature*. **554**, 255-259. https://doi.org/10.1038/nature25437

Marques, M. B., Ribeiro-Oliveira, A., Jr., Guimaraes, J., Nascimento, G. F., Anjos, A. P., Vilas-Boas, W. W., et al. (2012). Modifications in basal and stress-induced hypothalamic AMP-activated protein kinase (AMPK) activity in rats chronically treated with an angiotensin II receptor blocker. *Stress*. **15**, 554-561. https://doi.org/10.3109/10253890.2011.648673

Ohira, M., Endo, K., Saiki, A., Miyashita, Y., Terai, K., Murano, T., et al. (2012). Atorvastatin and pitavastatin enhance lipoprotein lipase production in L6 skeletal muscle cells through activation of adenosine monophosphate-activated protein kinase. *Metabolism*. **61**, 1452-1460. https://doi.org/10.1016/j.metabol.2012.03.010

Sag, D., Carling, D., Stout, R. D., Suttles, J. (2008). Adenosine 5'-monophosphate-activated protein kinase promotes macrophage polarization to an anti-inflammatory functional phenotype. *J Immunol*. **181**, 8633-8641. https://doi.org/10.4049/jimmunol.181.12.8633

Suarez-Martinez, E., Husain, K., Ferder, L. (2014). Adiponectin expression and the cardioprotective role of the vitamin D receptor activator paricalcitol and the angiotensin converting enzyme inhibitor enalapril in ApoE-deficient mice. *Ther Adv Cardiovasc Dis*. **8**, 224-236. https://doi.org/10.1177/1753944714542593

Sun, L., Yan, S., Wang, X., Zhao, S., Li, H., Wang, Y., et al. (2017). Metoprolol prevents chronic obstructive sleep apnea-induced atrial fibrillation by inhibiting structural, sympathetic nervous and metabolic remodeling of the atria. *Sci Rep*. **7**, 14941. https://doi.org/10.1038/s41598-017-14960-2

Sun, Y., Peng, X., Li, Y., Ma, H., Li, D., Shi, X. (2019). The effects of histamine H1 type receptor (H1R) in regulating osteoblastic cell differentiation and mineralization. *Artif Cells Nanomed Biotechnol*. **47**, 1281-1287. https://doi.org/10.1080/21691401.2019.1596924

Sung, J. Y., Choi, H. C. (2012). Nifedipine inhibits vascular smooth muscle cell proliferation and reactive oxygen species production through AMP-activated protein kinase signaling pathway. *Vascul Pharmacol*. **56**, 1-8. https://doi.org/10.1016/j.vph.2011.06.001

Tieu, B. C., Lee, C., Sun, H., Lejeune, W., Recinos, A., 3rd, Ju, X., et al. (2009). An adventitial IL-6/MCP1 amplification loop accelerates macrophage-mediated vascular inflammation leading to aortic dissection in mice. *J Clin Invest*. **119**, 3637-3651. https://doi.org/10.1172/JCI38308

Yang, H., Zhao, P., Tian, S. (2016). Clopidogrel Protects Endothelium by Hindering TNFalpha-Induced VCAM-1 Expression through CaMKKbeta/AMPK/Nrf2 Pathway. *J Diabetes Res*. **2016**, 9128050. https://doi.org/10.1155/2016/9128050
