# Supplementary figures and images for: Glycan Biosynthesis Ability of Gut Microbiota Increased in Primary Hypertension Patients Taking Antihypertension Medications and Potentially Promoted by Macrophage-Adenosine Monophosphate-Activated Protein Kinase
Source: Front Microbiol. 2021 Nov 4;12:719599. doi: 10.3389/fmicb.2021.719599 (PMC8600050; doi:10.3389/fmicb.2021.719599)

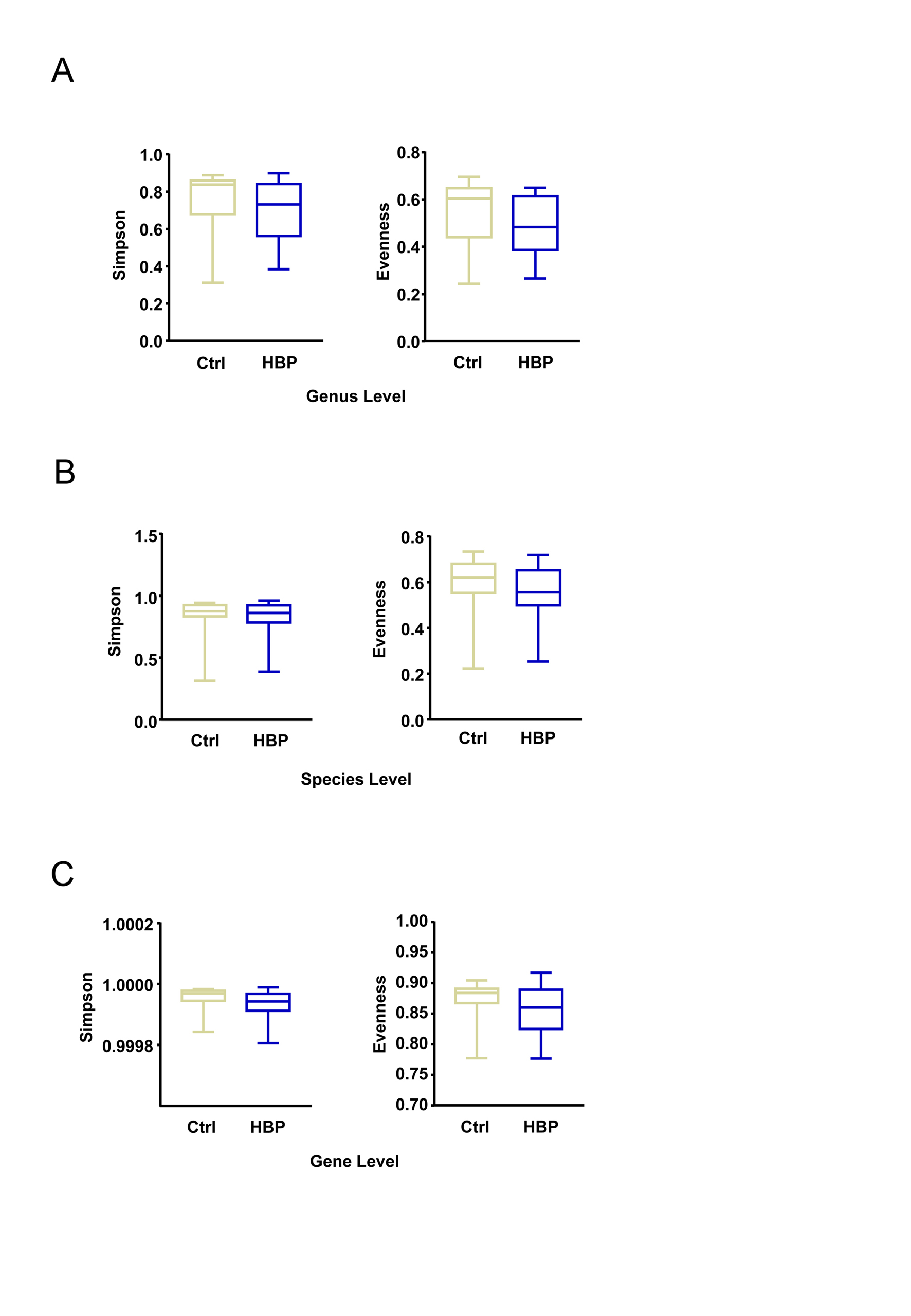

Supplement: Supplementary file 3 [file Image_1.JPEG]

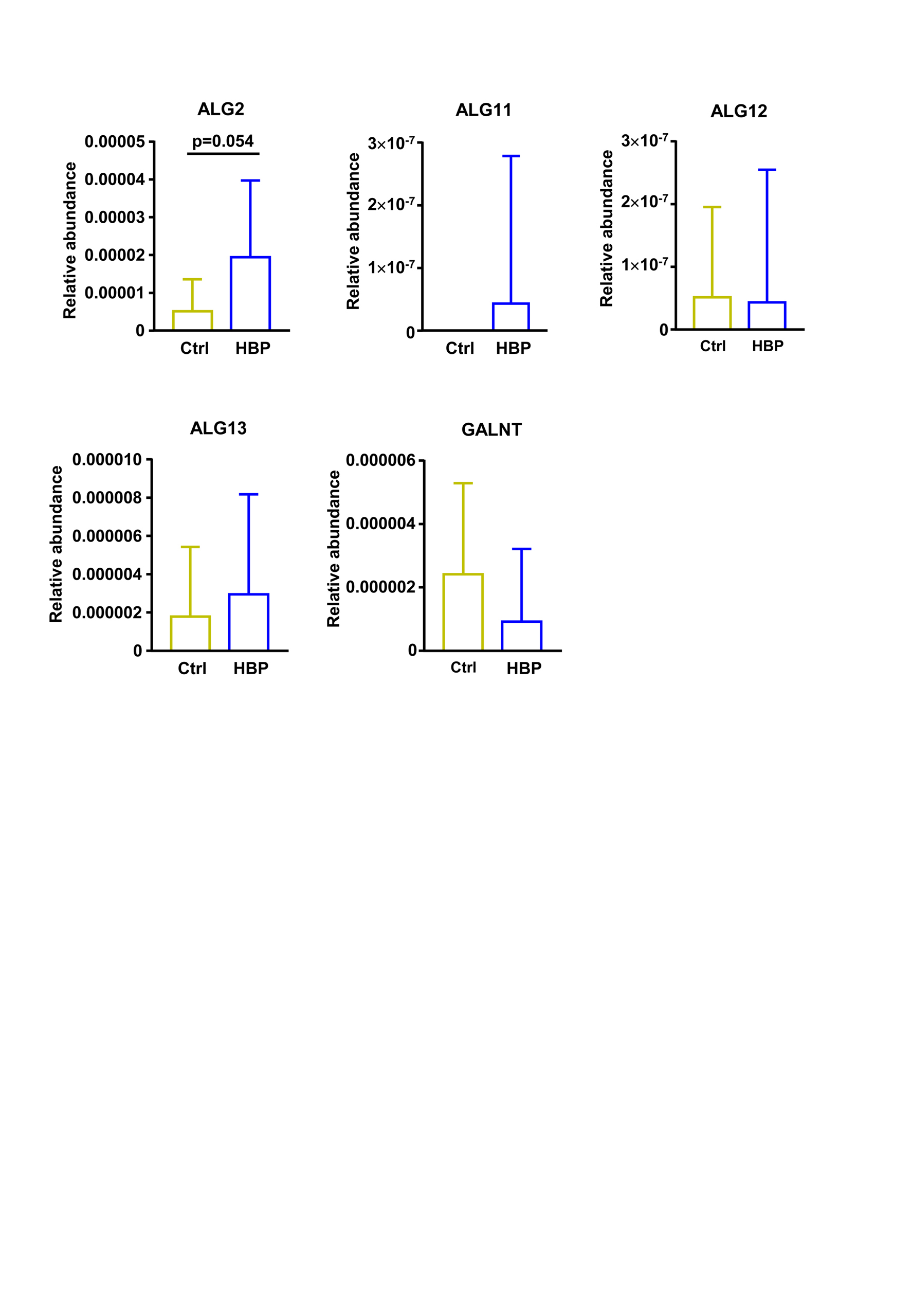

Supplement: Supplementary file 4 [file Image_2.JPEG]

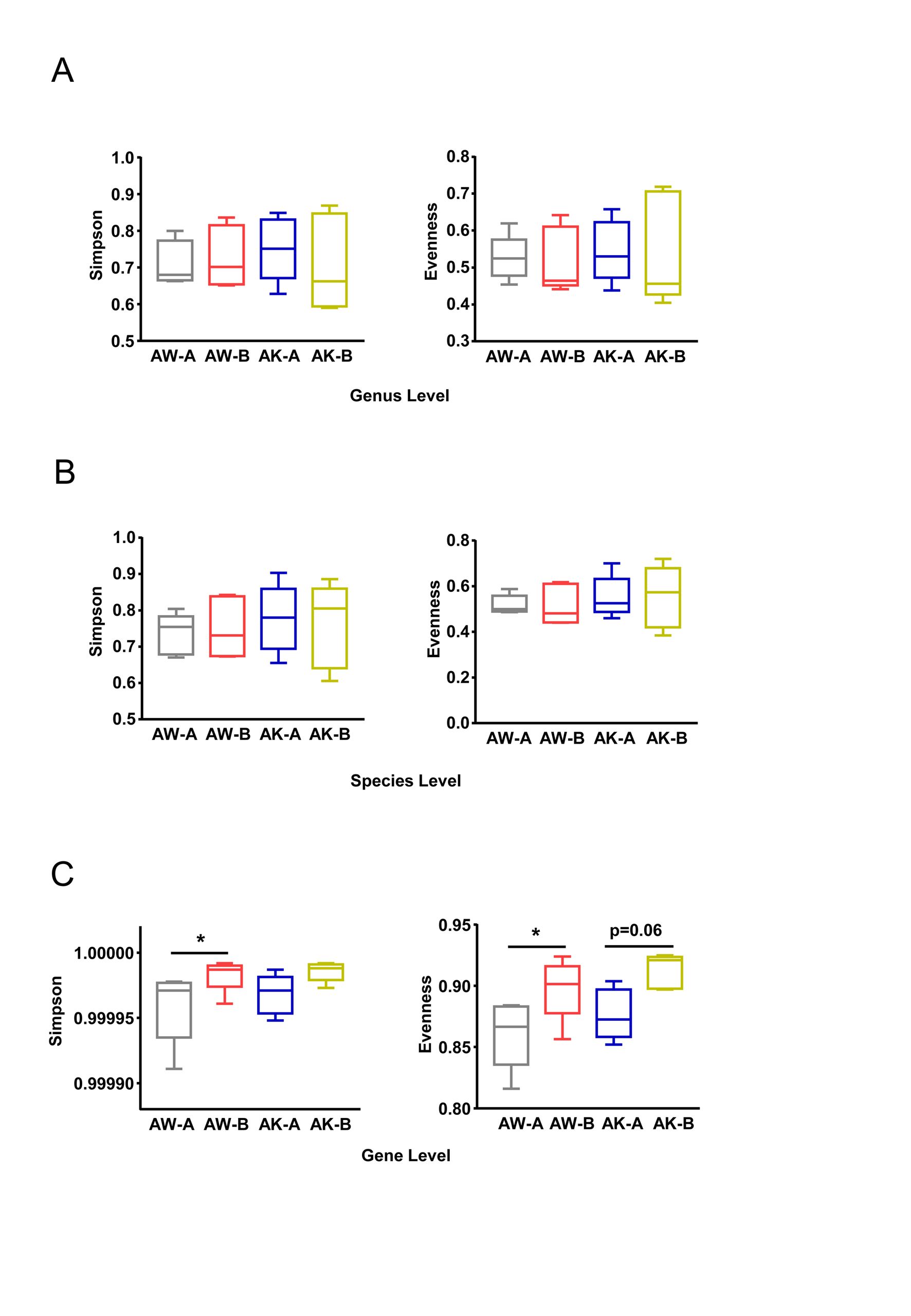

Supplement: Supplementary file 5 [file Image_3.JPEG]
